# Monozygotic Twins with MAGT1 Deficiency and Epstein–Barr virus-positive Classic Hodgkin Lymphoma Receiving anti-CD30 CAR T-cell Immunotherapy: A case Report

**DOI:** 10.1007/s10875-024-01690-0

**Published:** 2024-04-05

**Authors:** Jiachen Wang, Mi Zhou, Jianfeng Zhou, Min Xiao, Liang Huang

**Affiliations:** 1 Department of Hematology, Tongji Hospital, Tongji Medical College, Huazhong University of Science and Technology, Wuhan, China; 2 Immunotherapy Research Center for Hematologic Diseases of Hubei Province, Wuhan, China

## To the Editor,

Epstein-Barr virus (EBV)-positive classical Hodgkin lymphoma (cHL) is the most common malignancy in patients with “X-linked immunodeficiency with magnesium defect, EBV infection, and neoplasia” (XMEN). Aberrant expression of MAGT1 leads to XMEN, a congenital disorder of combined primary immunodeficiency (PID) characterized by increased susceptibility to chronic EBV infection and EBV-associated lymphoproliferation [1]. Patients with variants in *MAGT1* suffer from an N-linked glycosylation defect, resulting in low CD4^+^ cell counts with an inverted CD4:CD8 ratio, reduced expression of NKG2D (a natural killer (NK)-cell activating receptor) on NK cells and cytotoxic T lymphocytes (CTLs), and impaired T-cell activation through NKG2D [2].

A variety of approaches have been used to control this disease. However, the therapeutic effects are limited [3]. A new approach involving chimeric antigen receptor (CAR) T cells specific for CD30 can be used to treat relapsed or refractory (r/r) HL. Previously, we reported the safety, efficacy and robust long-term performance of CD30 CAR T-cell immunotherapy in our center [4]. However, there have been no reports on CAR T-cell therapy for lymphoma in XMEN thus far. Here, we present the first case of an identical twin with MAGT1-deficient cHL receiving murine CD30 CAR T-cell therapy and hope to provide insight into a therapeutic strategy for such patients.

## Case Presentation

The monozygotic twins were diagnosed with the cHL (mixed cellularity) at three and nine years of age. Twin 1 and twin 2 developed progressive disease (PD) after receiving chemotherapy, autologous HSCT, anti-CD30 antibodies, and anti-PD-1 monoclonal antibodies. *Supplemental Table 1* shows the therapeutic clinical therapy and disease state timeline of this case.

**Fig. 1:**
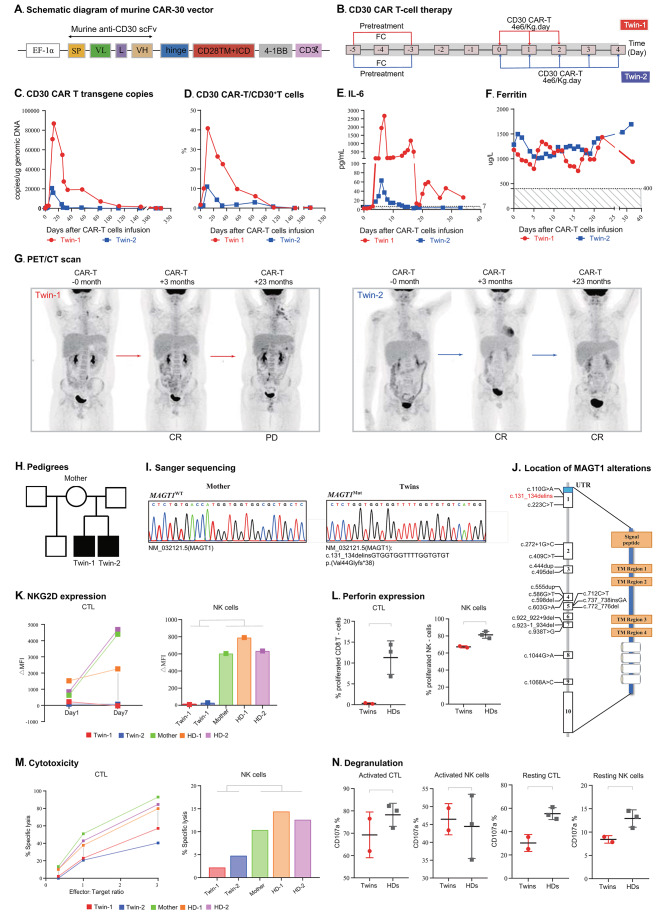
Clinical examination of the response to the infusion of murine anti-30 CAR T cell therapy. **(A)** Schematic diagram of the murine CAR30 vectors. The third-generation CARs were composed of a single chain variable fragment (scFv), two costimulatory domains from CD28 and 4-1BB, and CD3ζ chain as the activation domain. The scFv was derived from a murine monoclonal antibody against human CD30. ***Abbreviations***: SP, signal peptide; VL, variable L chain; L, linker; VH, variable H chain. **(B)** Lymphodepleting chemotherapy regimen and CAR T-cell infusion dose used in this case. Twin 1 and Twin 2 are represented in red and blue, respectively. **(C)** CAR30 transgene copy numbers detected by droplet digital polymerase chain reaction (ddPCR). **(D)** CAR30^+^ T-cell percentages among CD3^+^T cells detected by flow cytometry. **(E)** Levels of IL-6 after CAR T-cell therapy. **(F)** Levels of ferritin after CAR T-cell therapy. **(G)** FDG-PET/CT images obtained at 0 (left), + 3 months (middle), and + 20 months (right) after murine anti-CD30 CAR T-cell immunotherapy. Twin 1 and Twin 2 are represented in red and blue, respectively. **(H)** Pedigrees showing the families of the affected individuals (twins) harboring the *MAGT1* alteration. Solid symbols indicate affected persons who were hemizygous for the mutant allele; solid center symbols indicate unaffected persons hemizygous for the mutant allele; circles indicate female family members; squares indicate male family members. **(I)** Sanger sequencing of the region surrounding the missense *MAGT1* mutation (c.131_134delinsGTGGTGGTTTTGGTGTGT, p.Val44Glyfs*38) in a reference control subject and the patients. **(J)** Location of *MAGT1* variants in previously reported cases (represented in black) and in our patients (represented in red). **(K)** NKG2D protein expression in activated CD8^+^ T cells and activated CD56^+^ NK cells in twin-1, twin-2, mother and HDs, as measured by flow cytometry. **(L)** Perforin release in activated CD8^+^ T cells, and activated CD56^+^ NK cells in the twin-1, twin-2, mother and HDs, demonstrating markedly reduced expression in the twins’ cells, as measured by flow cytometry, was markedly reduced. **(M)** NK-cell cytotoxicity was measured by an enhanced green fluorescent protein (EGFP)-K562 flow cytometric method, and T-cell cytotoxicity was measured by CD8^+^ T cells against the Nalm6-luciferase cell line, which demonstrated markedly reduced NK-cell and T-cell cytotoxicity in the twins. **(N)** Degranulation was measured by flow cytometry in CD8^+^ T cells stimulated with CD3/CD28 Dynabeads and NK cells stimulated with the K562 cell line through CD107a expression, demonstrating defect in the degranulation of CD8^+^ T cells and NK cells in the twins. The Supplemental Materials and Methods section contains the contain details of the methodology. Due to the limited number of primary specimens, experiments in K.L.M.N. was performed only once

The twins were referred to our hospital to receive murine anti-CD30 CAR T cell therapy. Before treatment, CD30 target antigen expression was confirmed by immunohistochemical staining of the initially diagnosed lymph nodes (Supplemental Fig. 1). The structure (Fig. 1A) and manufacturing of CAR T cells are described in the Supplemental Methods as previously described [4]. The twins were given a standard dose of the FC regimen on days − 5 to -3 as lymphodepletion. Anti-CD30 CAR T cells (4 × 10^6^ kg/day) were infused on days 0–3 and days 0–5 for twins 1 and 2, respectively (Fig. 1B). T cells with the anti-CD30 CAR transgene expanded and persisted well in the twins compared with the previously reported average level in our center [4]. The key factors related to the therapeutic effect of CAR-T cells are presented in Supplemental Table 2. Compared to those of twin 2, twin 1 had a higher level of CAR T-cell expansion and longer persistence (Fig. 1C and D, Supplemental Table 3), and the level of interleukin-6 (IL-6) was greater in twin 1 (Fig. 1E). Grade 1 and grade 0 cytokine release syndrome (CRS) were observed in twin 1 and twin 2, respectively (Fig. 1F). Neither of the twins experienced any significant infections or neurological symptoms before or after CAR-T cell therapy. The twins achieved complete remission (CR) at + 3 months after CAR T-cell infusion. Twenty-three months later, twin 1 developed PD, but twin 2 remained in CR (Fig. 1G). Then, twins 1 and 2 received 200 mg of anti-PD-1 antibody every 1–2 months and every 2 months, respectively. As of December 2023, Twin-1 was maintained in stable disease (SD), and Twin-2 remained in CR.

In contrast, the twins’ half-brother, who shares the same mother and is 10 years older, currently remain healthy. The patients did not have a family history of cancer. To explore potential inborn errors of immunity (IEIs) underlying the persistent refractoriness of these young twins, whole-genome sequencing (WGS) was retrospectively performed on the peripheral blood mononuclear cells (PBMCs) of the twins and their older half-brother. WGS data have been uploaded to the National Center for Biotechnology Information (NCBI): PRJNA809536. The 2022 Immunological Societies Expert Committee (IUIS) updated gene list was included in the analysis. The filtered nonsynonymous variants are showed in Supplemental Table 4. Alterations that the twins shared but the half-brother lacked were selected. Among these, a complex hemizygous frameshift variant in *MAGT1* (c.131_134delinsGTGGTGGTTTTGGTGTGT, p.Val44Glyfs*38, NM_032121.5), which has never been reported in public databases (1000G, ExAC, and GenomAD), was identified (Fig. 1J). Sanger sequencing confirmed that the twins were hemizygous for the *MAGT1* frameshift variant, while their half-brother and mother carried the wild-type gene (Fig. 1H, I). In addition, XMEN-related clinical manifestations and laboratory findings of the twins were identified [1] and shown in Supplemental Fig. 2A-3 H.

Furthermore, several functional experiments in vitro were performed to explore the characteristics of XMEN. As the CAR30 transgene was detected at zero copy number by droplet digital polymerase chain reaction (ddPCR) in both twins, there was no impact on the activity of CAR T-cells in vitro. First, NKG2D, the best biomarker of XMEN disease, was assessed, and it was found to be decreased in both CD8^+^ T cells and NK cells from twins compared those from mothers and two healthy donors (Fig. 1K, Supplemental Fig. 3). Second, the twins’ CD8^+^ T cells and NK cells had impaired expression of perforin (Fig. 1L, Supplemental Fig. 4) and diminished cytotoxicity when stimulated (Fig. 1M). Third, degranulation assays indicated that activated CTLs and NK cells were normal, while resting CTLs and NK cells were deficient (Fig. 1N, Supplemental Fig. 5).

## Discussion and Conclusions

XMEN is caused by loss-of-function variants in MAGT1. In the present case, identical twins suffered from the same type of disease due to the same *MAGT1* hemizygous deletion. In addition, next-generation sequencing (NGS) revealed typical genetic aberrations (Supplemental Table 4) in cHL according to the initial diagnosis via formalin-fixed, paraffin-embedded (FFPE) sequencing, indicating that germline *MAGT1* alteration was the pathogenic driving factor [5].

In the present patients, *MAGT1* germline mutation screening was not performed during diagnosis or at the beginning of CAR T-cell therapy. Our case underscores the importance of identifying *MAGT1* deficiency in young patients with EBV-positive lymphoproliferative disease through high-throughput sequencing.

There is no international consensus on the treatment of XMEN. Anti-CD20 therapy with rituximab for EBV control has not been recommended because of its inconsistent efficacy and lack of effect on chronic EBV infection [1]. Magnesium supplementation therapy has been proven ineffective in a clinical trial (US National Institutes of Health ClinicalTrials.gov#NCT02496676). HSCT has also been attempted in some patients, but posttransplant mortality remains high [3]. Recently, Brault et al. presented data on a novel gene-editing approach that utilizes CRISPR-Cas9 to compensate for the deletion of the *MAGT1* gene [6]. Despite this exciting progress, there is still a long way to go for clinical applications of this new technique.

This case provides evidence for the use of anti-CD30 CAR T-cell therapy in hematologic malignancy patients with germline *MAGT1* variants. Although XMEN patients had significant CTL dysfunction, both twins achieved CR after CAR T-cell immunotherapy (twin 1 developed PD twenty-three months later). We suspect that the CAR T-cell component might compensate for the T-cell defects, and that other cytotoxic mechanisms of CAR-T cell, such as cytokine-mediated killing (e.g., killing via IFNγ), may compensate for the perforin-deficient effects on cytotoxicity. Since this was a retrospective study, CAR T-cell functional experiments were not performed in vitro. It took twenty-three months for twin 1 to relapse, while twin 2 remained in CR. Compared to twin 2, twin 1 had a greater disease load, lower infusion dose of CAR T cells, stronger CAR transgene amplification, and greater CRS. This result suggested that a low tumor load and an adequate infusion dose of CAR T cells are necessary for prolonged CR.

Although allo-HSCT can be curative for immunodeficient patients, most XMEN patients die from transplant-related complications [3]. The decision for allo-HSCT in patients with XMEN, should be balanced against the risks and the availability of a suitable donor. In this case, we recommended that Twin-1 received allo-HSCT from an unrelated healthy donor as a salvage approach when the disease progressed after CAR T-cell therapy. However, his parents repeatedly declined HSCT and requested that he take anti-PD-1 antibodies for maintenance.

In conclusion, if lymphoma is diagnosed at a young age or has a poor therapeutic outcome, it should be suspected to be a possible IEI. A novel inherited germline alteration in MAGT1 was identified, and this case is the first time CAR T-cell immunotherapy has been used in XMEN. CD30 CAR T-cell therapy may be a viable option for XMEN patients with r/r HL. More prospective experimental data are needed to explore the potential of bridging HSCT with CAR T-cell therapy.

### Electronic Supplementary Material

Below is the link to the electronic supplementary material.

Supplementary Material 1

## Data Availability

The WGS datasets presented in this study have been uploaded to the NCBI under the accession number PRJNA809536 (https://www.ncbi.nlm.nih.gov/bioproject/PRJNA809536). The cytologic data of the patients can be available by contacting the corresponding author upon reasonable request.

## Abbreviations

1000G: 1000 Genomes Project
allo-HSCT: allogeneic hematopoietic stem cell transplantation
anti-PD-1: anti-programmed cell death protein 1 antibody
autoHSCT: autologous hematopoietic stem cell transplantation
Bio-RAID: Biology Research and Development,chimeric antigen receptor
CD107a: cluster of differentiation 107a
CD3ζ: CD3-zeta chain
ChiCTR: Chinese clinical trial registry
cHL: classic Hodgkin lymphoma
CR: complete remission
CRS: cytokine release syndrome
CTL: cytotoxic T lymphocyte
delins: deletion/insertion
ddPCR: droplet digital polymerase chain reaction
DNA: deoxyribonucleic acid
DNM: de novo mutation
Dr.: doctor
EBV: Epstein-Barr virus
EGFP: enhanced green fluorescent protein
FDG-PET/CT: fluorodeoxyglucose positron emission tomography/computed tomography
ExAC: Exome Aggregation Consortium
Prof: professor
fs: frame shift
FFPE: formalin-fixed,paraffin-embedded
GenomAD: Genome Aggregation Database
Gly: glycine
HD: healthy donor,IEI,Inborn Errors of Immunity
IFNγ: Interferon-gamma
IL-6: interleukin-6
IUIS: Union of Immunological Societies Expert Committee
MAF: minor allele frequency
MAGT1: magnesium transporter 1
NCBI: National Center for Biotechnology Information
NGS: next-generation sequencing
No: number
NK: natural killer
NKG2D: natural killer group 2,member D
PET/CT: positron emission tomography/computed tomography
PBMC: peripheral blood mononuclear cell
PD: progressive disease
PID: primary immunodeficiency
r/r: relapsed/refractory
SD: stable disease
scFv: single chain variable fragment
SP: signal peptide
TCR: T-cell receptor
VL: variable L chain
Val: Valine
VH: variable H chain
WGS: whole-genome sequencing
XMEN: X-linked immunodeficiency with magnesium defect,EBV infection,and neoplasia

## Declarations

### Trial Registration

This study received approval from the institutional review board of Tongji Hospital, Tongji Medical College, and Huazhong University of Science and Technology. It was registered with the Chinese Clinical Trial Registry (ChiCTR) under the registration number ChiCTR-OPN16009069.
