# Correction to: Monozygotic Twins with MAGT1 Deficiency and Epstein-Barr virus-positive Classic Hodgkin Lymphoma Receiving anti-CD30 CAR T-cell Immunotherapy: A case Report

**DOI:** 10.1007/s10875-024-01723-8

**Published:** 2024-05-25

**Authors:** Jiachen Wang, Mi Zhou, Jianfeng Zhou, Min Xiao, Liang Huang

**Affiliations:** 1 Department of Hematology, Tongji Hospital, Tongji Medical, College, Huazhong University of Science and Technology, Wuhan, China; 2 Immunotherapy Research Center for Hematologic Diseases of Hubei Province, Wuhan, China

**Correction to** **:** **Journal of Clinical Immunology**

10.1007/s10875-024-01690-0

Due to a formatting error, there was an overlap in the original Fig. 1 J.
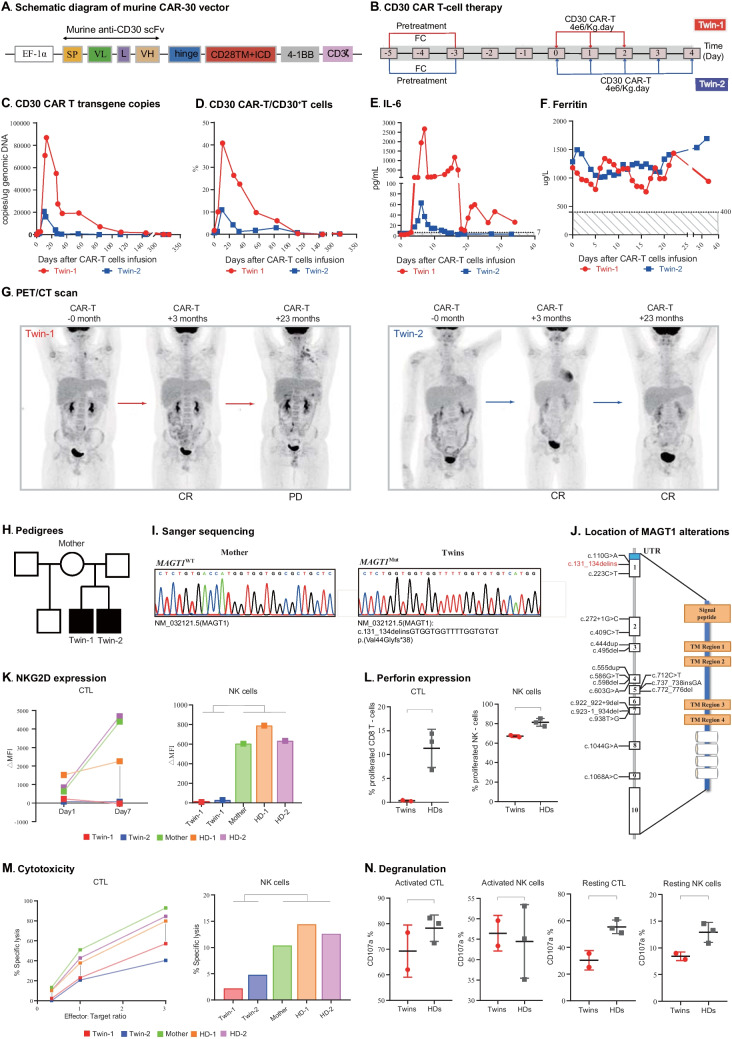


The original version has been corrected.

