# Supplementary material for: Monozygotic Twins with MAGT1 Deficiency and Epstein–Barr virus-positive Classic Hodgkin Lymphoma Receiving anti-CD30 CAR T-cell Immunotherapy: A case Report
Source: J Clin Immunol. 2024 Apr 5;44(4):91. doi: 10.1007/s10875-024-01690-0 (PMC10997540; doi:10.1007/s10875-024-01690-0)
Supplement: Supplementary file 1 — Supplementary Material 1 [file 10875_2024_1690_MOESM1_ESM.docx]

**Monozygotic twins with MAGT1 deficiency and Epstein–Barr virus-positive classic Hodgkin lymphoma receiving anti-CD30 CAR T-cell immunotherapy: A case report**

Supplementary Appendix

**Table of Contents**

**Supplementary Figures**

**Supplementary Tables**

**Supplementary Materials and Methods**

**Supplementary References**

##
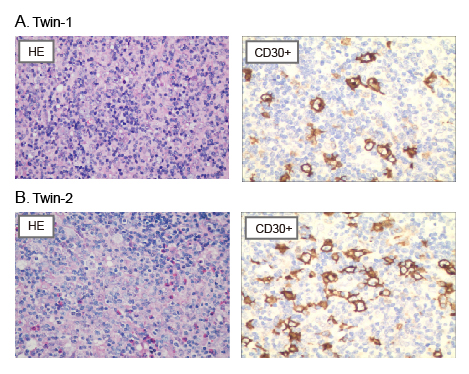
Supplemental Figures


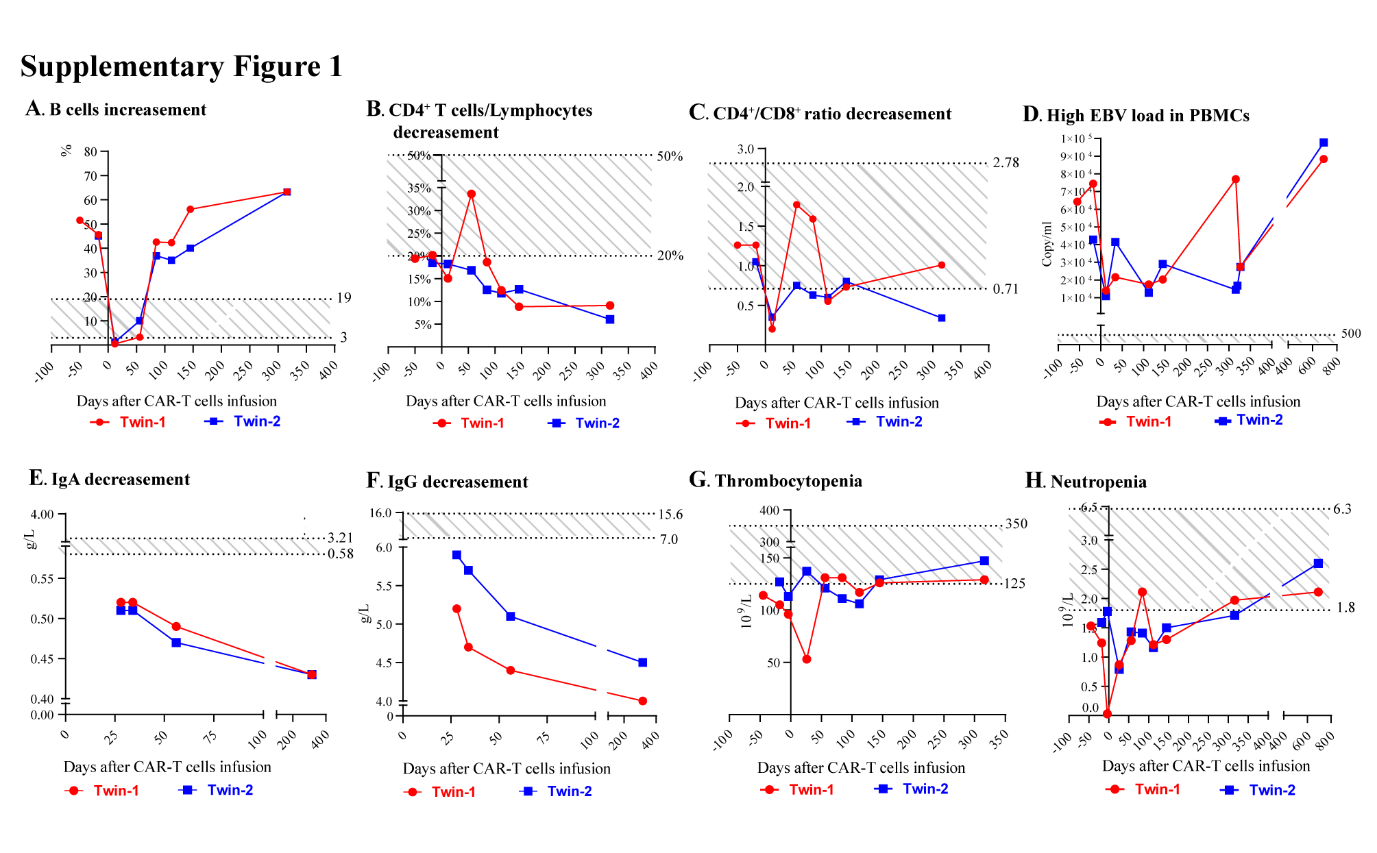
**Supplemental Fig. 1 Pathological and immunohistochemical staining of the initial diagnostic FFPE samples of the twins. (A)** H&E staining showing large pleomorphic Hodgkin-like cells mixed with lymphocytes and histiocytes. **(B)** Strong cytoplasmic CD30 expression in the multinucleated Reed–Sternberg cells of cHLs. In the background, normal lymphocytes remained unstained.

**Supplemental Fig. 2 Clinical examination of the response to infusion of murine anti-CD30 CAR T-cell therapy. The stippled areas represent the range of normal values.** Levels of CD19^+^ B cells/lymphocytes **(A)**, CD4^+^ T cells/lymphocytes **(B)**, CD4^+^ T/CD8^+^ T cells **(C)**, the EBV load in PBMCs **(D)**, IgA in plasma **(E)**, IgG in plasma **(F)**, blood platelets **(G)**, and neutrophil counts **(H)**
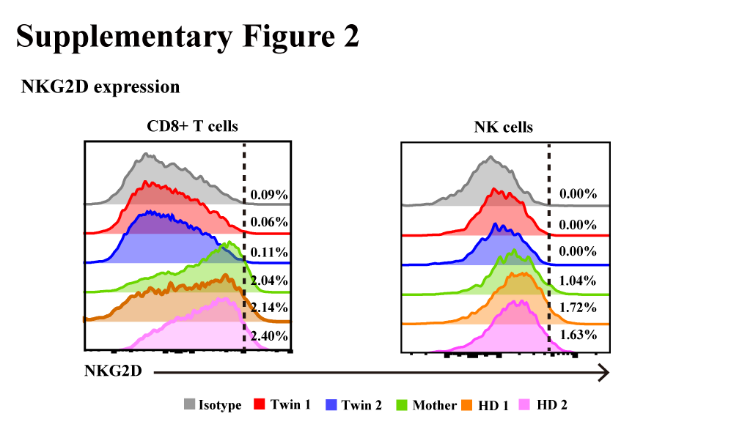
during anti-CD30 CAR T-cell therapy. Twin-1 and Twin-2 are represented in red and blue, respectively.

**Supplemental Fig.3 NKG2D expression in CD8^+^ T cells and NK cells.** Flow cytometry histograms of NKG2D protein expression in activated CD8^+^ T cells and activated CD3^-^CD56^+^ NK cells from the isotype controls, patients, mother, and HDs.


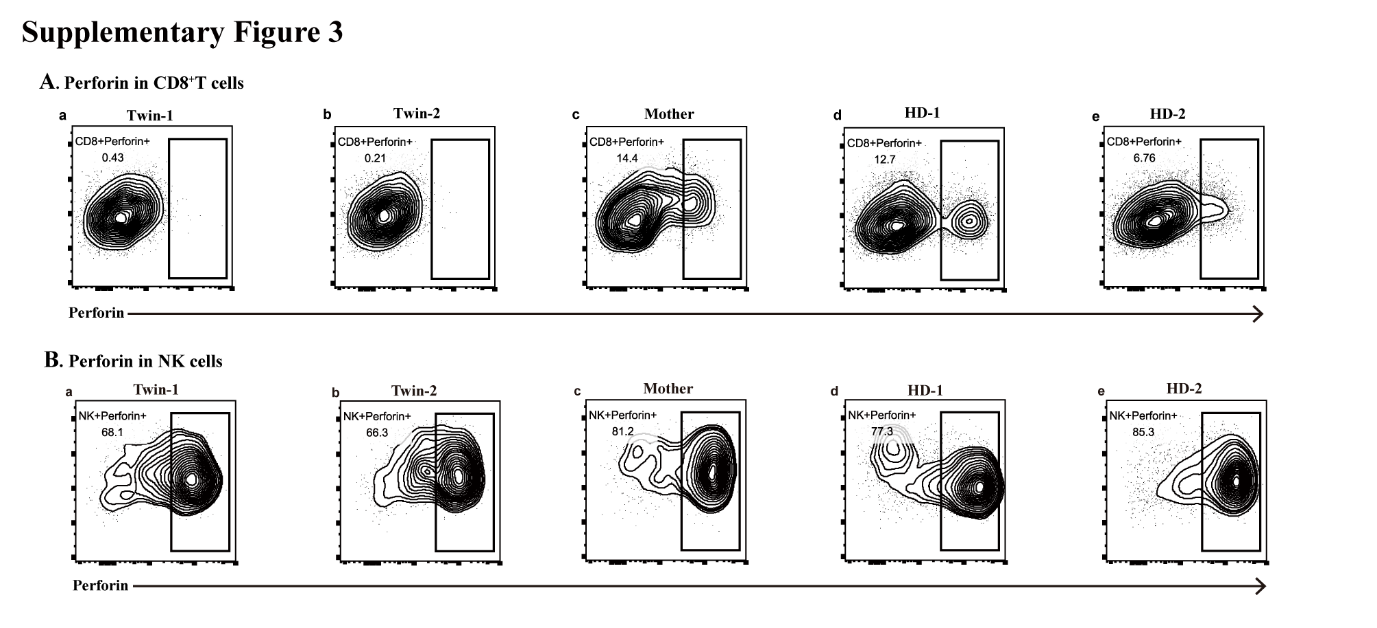


**Supplemental Fig.4 Perforin in CD8^+^ T cells and NK cells.** Flow cytometry histograms of perforin release in activated CD8^+^ T cells (A) and activated CD3^-^CD56^+^ NK cells (B) from patients, mother, and HDs.


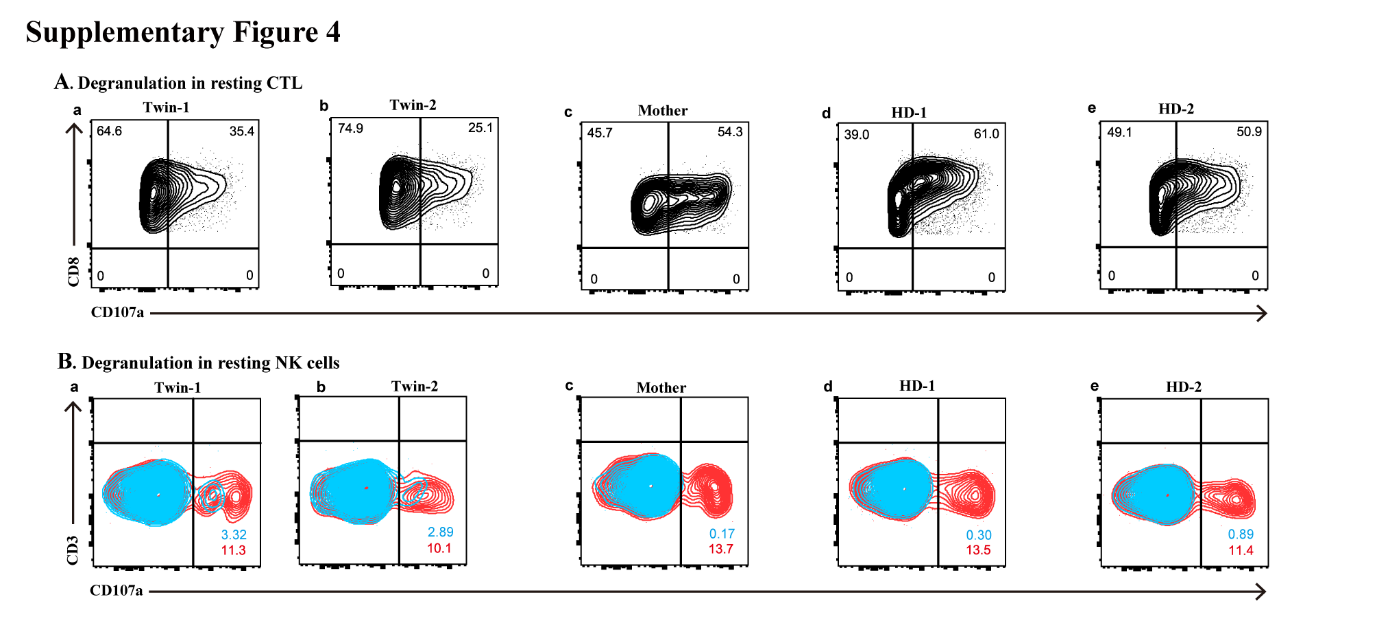


**Supplemental Fig.5 Degranulation in CD8^+^ T cells and NK cells.** Flow cytometry histograms of perforin release in activated CD8^+^ T cells and activated CD3^-^CD56^+^ NK cells from patients, mother, and HDs.

## Supplementary Tables

***Supplementary Table 1. The therapeutic timeline of clinical therapy and disease state in this patient***

**Twin-1**

| **Time since**  **CAR-T infusion.** | **Clinical therapy** | **Disease state** |
| --- | --- | --- |
| *-55 month* | Initial diagnosis of cHL (mixed cell type) | - |
| *-55 to -46 month* | Chemotherapy for 8 cycles (regimens for children with Hodgkin lymphoma in China) | CR (*-46 month*) |
| *-46 month* | AutoHSCT | - |
| *-45 to -13 month* | Regular follow-up | CR (*-13 month*) |
| *-1 year* | - | PD |
| *-11 to -8 month* | Brentuximab vedotin + Gemcitabine | PD (*-8 month*) |
| *-7 to -2 month* | PD-1 monoclonal antibody for 8 cycles | PD (*-2 month*) |
| *-5 to -3 day* | Pretreatment: Fludarabine + CTX | - |
| ***0 to +3 day*** | ***CD30 CAR T-cell infusion:***  ***4*10^6^/kg.day × 3 days*** | - |
| *+2 to +4 month* | 100 mg PD-1 monoclonal antibody each month | CR (*+3 month*) |
| *+5 to +10 month* | Regular PD-1 monoclonal antibody | PD (*+23 month*) |
| *+11 month until now* | 200 mg PD-1 monoclonal antibody every 1-2 months | SD (*+51 month*) |

**Twin-2**

| **Time since**  **CAR-T infusion.** | **Clinical therapy** | **Disease state** |
| --- | --- | --- |
| *-10 year* | Initial diagnosis of cHL (mixed cell type) | - |
| *-10 to -9 year* | Chemotherapy for 6 cycles (regimens for children with Hodgkin lymphoma in China) | CR |
| *-80 month* | - | PD (-*80 month*) |
| *-79 to -74 month* | Chemotherapy for 8 cycles (regimens for children with Hodgkin lymphoma in China) | CR |
| *-7 year* | AutoHSCT | - |
| *-7 to -4 year* | Regular follow-up | CR |
| *-4 year* | - | PD (-*4 year*) |
| *-4 to -1 year* | Chemotherapy for 4 cycles (regimens for children with Hodgkin lymphoma in China) | - |
| *-10 month* | - | PD |
| *-9 to -4 month* | Brentuximab vedotin + Gemcitabine for 8 cycles | PD (-*3 month*) |
| *-3 month to -4 day* | PD-1 monoclonal antibody for 4 cycles | PD (-*0.5 month*) |
| *-5 to -3 day* | Pretreatment: Fludarabine + CTX | - |
| ***0 to +5 day*** | ***CD30 CAR T-cell infusion:***  ***4*10^6^/kg.day × 5 days*** | - |
| *+1 to +4 month* | 100 mg PD-1 monoclonal antibody each month | CR (*+3 month*) |
| *+5 to +10 month* | Regular PD-1 monoclonal antibody | CR (*+23 month*) |
| *+11 month until now* | 200 mg PD-1 monoclonal antibody every 2 months | CR (*+51 month*) |

Twin 1 achieved complete remission (CR) after nine cycles of chemotherapy regimens for pediatric cHL and subsequently underwent autologous hematopoietic stem cell transplantation (autoHSCT). After 32 months of autoHSCT, twin 1 developed progressive disease (PD). However, Twin 1 still developed progressive disease (PD) after anti-CD30 antibody plus decitabine regimens and eight cycles of anti-PD1 antibody therapy at age fourteen. Meanwhile, twin 2 received six cycles of chemotherapy for pediatric cHL to achieve a CR at age three and developed PD at age six. Twin 2 then received another eight cycles of chemotherapy to achieve CR, followed by autoHSCT At ten years of age, twin 2 developed PD again. Eight cycles of the anti-CD30 antibody plus decitabine regimen and four cycles of the anti-PD1 antibody therapy were used, and twin 2 patients still developed PD.

***Supplementary Table 2. Key factors related to CAR T-cell therapeutic effects***

|  | **Twin-1** | **Twin-2** |
| --- | --- | --- |
| **Disease status** |  |  |
| Before CAR T-cell infusion | PD | PD |
| After CAR T-cell infusion | CR (month +3) | CR (month +3) |
|  | PD (month +17) | CR (month +17) |
|  | SD (until now) | CR (until now) |
| **Disease burden** |  |  |
| SUVmax in PET/CT | 3.7 ↑ | 2.4 ↓ |
| LDH (U/L) | 201 | 206 |
| **Factors related to CAR Tcells** |  |  |
| Pretreatment regimen | FC day1-3  (*F:25 mg/m^2^, C:20 mg/kg*) | FC day 1-3  (*F:25 mg/m^2^, C:20 mg/kg*) |
| CAR T-cell infusion dose | 4*10^6^/kg.day × 3 days ↓ | 4*10^6^/kg.day × 5 days ↓ |
| Level of CRS grade | One ↑ | Zero ↓ |
| CAR transgene amplification (C_max_) | 86869 copies/μg DNA↑ | 20661 copies/μg DNA↓ |
| CAR transgene persistence (T_max_) | +316 day ↑ | +145 day ↓ |
| **Therapy after CAR T-cell infusion** |  |  |
| Sintilimab injection (an anti-PD-1 antibody) | Once every one to two months | Once every two months |

***Supplementary Table 3. CD30 CAR T transgene copies***

| Days after CAR T-cell infusion  (day) | Twin-1  (copies/μg DNA) | Twin-2  (copies/μg DNA) |
| --- | --- | --- |
| 0 | 0 | 0 |
| 1 | 830 | 217 |
| 2 | 2419 | 748 |
| 5 | 2696 | 1561 |
| 11 | 70890 | 20661 |
| 13 | 86869 | 15993 |
| 26 | 54805 | 4192 |
| 28 | 27483 | 803 |
| 34 | 18953 | 511 |
| 56 | 19313 | 742 |
| 84 | 7027 | 180 |
| 112 | 2289 | 83 |
| 145 | 1630 | 178 |
| 316 | 220 | 0 |
| 327 | 0 | 0 |

***Supplementary Table 4. The filtered nonsynonymous variants in the IUIS-2022 gene list of the patient with a minor allele frequency of < 0.001 in gnomAD discovered by WGS.***

**Twin-1**

| Gene | Variant | NM | Variant_  Frequency | COSMIC | dbSNP | Allele_Frequency_  popmax |
| --- | --- | --- | --- | --- | --- | --- |
| MAGT1 | c.130_131insGTGGTGGTTTTGGTG  (p.Ser43_Val44insGlyGlyGlyPheGly) | NM_032121.5 | 100.00% | . |  | 0 |
| C2orf69 | c.1080G>A(p.Met360Ile) | NM_153689.6 | 60.60% | . | . | 0 |
| C5 | c.3426A>C(p.Leu1142Phe) | NM_001735.2 | 46.70% | . | . | 0 |
| PRF1 | c.503G>A(p.Ser168Asn) | NM_001083116.3 | 45.90% | 4382352 | rs779399414 | 0.0018 |

**Twin-2**

| Gene | Variant | NM | Variant_  Frequency | COSMIC | dbSNP | Allele_Frequency_  popmax |
| --- | --- | --- | --- | --- | --- | --- |
| MAGT1 | c.130_131insGTGGTGGTTTTGGTG  (p.Ser43_Val44insGlyGlyGlyPheGly) | NM_032121.5 | 100.00% | . | . | 0 |
| C5 | c.3426A>C(p.Leu1142Phe) | NM_001735.2 | 49.00% | . | . | 0 |
| CHD7 | c.8605T>C(p.Ser2869Pro) | NM_017780.4 | 47.70% | . | rs778436403 | 0.0003 |
| LYST | c.3310C>T(p.Arg1104Ter) | NM_001301365.1 | 43.20% | . | rs80338652 | 6.06E-05 |
| MOGS | c.1693C>T(p.Arg565Trp) | NM_006302.3 | 43.10% | . | rs745841543 | 1.50E-05 |

**Half-brother**

| Gene | Variant | NM | Variant_  Frequency | COSMIC | dbSNP | Allele_Frequency_  popmax |
| --- | --- | --- | --- | --- | --- | --- |
| LYST | c.3310C>T(p.Arg1104Ter) | NM_001301365.1 | 52.90% | . | rs80338652 | 6.06E-05 |
| ITGB2 | c.598C>T(p.Pro200Ser) | NM_000211.5 | 52.20% | . | rs756755210 | 0.0003 |
| ALPI | c.1324G>T(p.Ala442Ser) | NM_001631.5 | 43.20% | . | . | 0 |

***Supplementary Table 5. List of genes in the targeted NGS panel (n=157)***

| *ABL1* | *ACTG1* | *AKT1* | *ARID1A* | *ATM* | *ATP6AP1* | *ATP6V1B2* |
| --- | --- | --- | --- | --- | --- | --- |
| *B2M* | *BCL10* | *BCL11B* | *BCL2* | *BCL6* | *BCOR* | *BIRC3* |
| *BRAF* | *BTG1* | *BTG2* | *BTK* | *CARD11* | *CASP10* | *CCND1* |
| *CCND3* | *CCR4* | *CCR7* | *CD28* | *CD58* | *CD70* | *CD79A* |
| *CD79B* | *CD83* | *CDKN1B* | *CDKN2A* | *CHD2* | *CNOT3* | *CREBBP* |
| *CRLF2* | *CTNNB1* | *CXCR4* | *DDX3X* | *DIS3* | *DNM2* | *DNMT3A* |
| *DTX1* | *DUSP2* | *EBF1* | *EED* | *EGR1* | *EGR2* | *EIF2A* |
| *EP300* | *ETV6* | *EZH2* | *FAM46C* | *FAS* | *FAT1* | *FBXW7* |
| *FGFR3* | *FLT3* | *FOXO1* | *FYN* | *GATA3* | *GNA13* | *GNAQ* |
| *GPR183* | *HIF1A* | *HIST1H1B* | *HIST1H1C* | *HIST1H1D* | *HIST1H1E* | *HNRNPA2B1* |
| *HRAS* | *HVCN1* | *ID3* | *IDH1* | *IDH2* | *IGLL5* | *IKBKB* |
| *IKZF1* | *IKZF3* | *IL7R* | *IRF4* | *ITPKB* | *JAK1* | *JAK2* |
| *JAK3* | *KDM6A* | *KIT* | *KLF2* | *KLHL6* | *KMT2C* | *KMT2D* |
| *KRAS* | *LTB* | *MAP2K1* | *MAP3K14* | *MAPK1* | *MAX* | *MED12* |
| *MEF2B* | *MYC* | *MYD88* | *NF1* | *NFE2* | *NFKBIE* | *NOTCH1* |
| *NOTCH2* | *NRAS* | *NT5C2* | *PAX5* | *PHF6* | *PIK3CA* | *PIK3R1* |
| *PIM1* | *PLCG1* | *PLCG2* | *POT1* | *POU2AF1* | *POU2F2* | *PRDM1* |
| *PRKCB* | *PTEN* | *PTPN1* | *PTPN11* | *RB1* | *RHOA* | *RPL10* |
| *RPS15* | *RRAGC* | *SAMHD1* | *SETD2* | *SF3B1* | *SGK1* | *SH2B3* |
| *SMARCA4* | *SMARCB1* | *SOCS1* | *STAT3* | *STAT5B* | *STAT6* | *TBL1XR1* |
| *TCF3* | *TET1* | *TET2* | *TMSB4X* | *TNFAIP3* | *TNFRSF14* | *TNFRSF1B* |
| *TP53* | *TRAF3* | *TRRAP* | *U2AF1* | *USP7* | *VAV1* | *VMA21* |
| *WHSC1* | *WT1* | *XPO1* |  |  |  |  |

## Supplementary Materials and Methods

***Study Subjects***

All procedures were performed upon informed consent and assent from patients, first-degree relatives, and healthy donor controls in accordance with the ethical standards of the institutional and/or national research committees and with the current update of the Declaration of Helsinki.

***Clinical procedures***

As referred to previously published articles from our center(1, 2), eligible patients received hematopoietic stem cell (HSC) mobilization and apheresis for ASCT and lymphocyte apheresis to obtain adequate lymphocytes from peripheral blood for chimeric antigen receptor (CAR) T-cell manufacturing. The CAR used in this trial is a third-generation CAR composed of a single-chain variable fragment (scFv) targeting CD30, two costimulatory domains from CD28 and 4-1BB, and a CD3ζ chain as the activation domain (2). Validation of the CAR constructs and procedures for cell production and quality control assays have been described previously (2). Assessment of T-cell subsets of CAR30 T-cell products was performed as previously described (3).

***Laboratory assessments***

For patients who had bone marrow involvement, multiparameter flow cytometry (MFC) was used to quantitate minimal residual disease. In vivo expansion of CAR30-expressing T cells was measured by droplet digital polymerase chain reaction (ddPCR), detailed procedure used was previously described (4).

***Targeted high-throughput sequencing of formalin-fixed paraffin-embedded (FFPE)***

A total of 157 genes were selected for this study (Supplementary Table S4). Using the hg19/GRCh37 genome as a reference, a sequencing panel covering the coding sequences (CDSs) in 157 genes was designed online (Design studio Sequencing, Illumina, San Diego, USA). Sequencing libraries were prepared with AmpliSeq™ Library PLUS for Illumina, using 20 ng of input genomic DNA per sample. Library sequencing was performed to 2000× coverage on a NextSeq™ 550 system using an Illumina NextSeq™ 500/550 High Output v2 Kit (Illumina, San Diego, USA). Alignments and variant calling were performed using a DNA Amplicon workflow with default parameters on a BaseSpace Sequence Hub (Illumina). The generated variants were further annotated using ANNOVAR. The detailed procedure used for variant filtering was as previously described.(5)

***Genetic analysis***

For whole‐exome sequencing (WES) of the initial bone marrow sample, a library was constructed using a Fast Library Prep Kit, after which whole exons were identified with an AIExome Enrichment Kit V1 (iGeneTech). Sequencing was performed on the Illumina platform (Illumina) with 150–base‐paired‐end reads. The raw reads were filtered to remove low‐quality reads using FastQC. Then, the clean reads were mapped to the reference genome GRCh37 using BWA. After removing duplications, SNVs and InDels were called and annotated using GATK. The sequence variants are described in the HGVS nomenclature. MAGT1 mutations were confirmed by Sanger sequencing as follows: forward primer, CGGACCAATGAAAACGCTCC; reverse primer, GGCTGTCACCTACGCTACTC; and product length, 560 bpm.

***Expansion of T cells and NK cells***

Fresh whole‐blood samples were collected in EDTA‐containing vials and sent to the laboratory at room temperature. PBMCs were isolated by standard Ficoll–Hypaque gradient centrifugation as previously described (6). T cells were expanded by stimulating PBMCs with Dynabeads™ Human T‐Activator CD3/CD28 (Gibco, Grand Island, NY, USA) at a 1:5 ratio in CTS™ OpTmizer™ medium (Gibco, USA) supplemented with 10% fetal bovine serum (FBS, Gibco, USA), 2 mM l‐glutamine (Gibco, USA) and 200 IU mL−1 rhIL‐2 (PeproTech, Rocky Hill, NJ, USA). NK cells were expanded by stimulating the PBMCs with 100 IU mL−1 rhIL‐2 (PeproTech, USA).

***Flow cytometry and antibodies***

For analysis of cell surface markers, PBMCs were used as the starting material. The cells were washed twice in FACS buffer (PBS with 5% FBS) and resuspended in 100 μl of FACS buffer supplemented with antibodies for 30 min on ice. The cells were washed twice in FACS buffer and resuspended in 300 μl of FACS buffer. The data were acquired on a FACS Calibur (Becton Dickinson) and analyzed using the FlowJo software (TreeStar).

***NKG2D expression***

NKG2D expression on T cells was measured upon 48‐h stimulation of PBMCs with Dynabeads™ Human T‐Activator CD3/CD28 (Gibco, USA) at a 1:5 ratio. The cells were stained with anti‐CD314(NKG2D)‐APC/CY7 (clone *1D11*, BioLegend, San Diego, USA), anti‐CD3‐PacB (clone *SK7*, BioLegend, USA), anti‐CD4‐BV605 (clone *RPA-T4*, BD Biosciences, USA) and anti‐CD8‐PE‐Cy7 (clone *SK1*, BioLegend, USA) antibodies. The expression on activated NK cells and B cells was measured after 48 h of stimulation of PBMCs with 600 U/mL IL‐2 (Peprotech, USA). PBMCs were stained with anti‐CD314(NKG2D)‐APC/CY7 (clone *1D11*, BioLegend, USA), anti‐CD3‐PacB (clone *SK7*, BioLegend, USA), and anti‐CD56‐PE (clone *B159*, BD Biosciences, USA) antibodies.

***Perforin-mediated intracellular staining***

For each staining, 2 × 10^5^ PBMCs on day 0 were washed and surface stained with anti‐CD3‐Percp (clone *SK7*, BD Biosciences, USA), anti‐CD8‐APC‐Cy7 (clone *SK1*, BioLegend, USA) and anti‐CD56‐APC (clone *HCD56*, BioLegend, USA) in FACS buffer. For intracellular staining, cells were fixed and permeabilized with Cytofix/Cytoperm solution (BD Biosciences, USA) according to the manufacturer's instructions and stained intracellularly with anti‐perforin‐PE (clone *B‐D48*, BioLegend, USA).

***CTL and NK cell degranulation tests***

NK cell and CTL (cytotoxic T lymphocyte) degranulation were assessed by CD107a surface staining without medium‐cultured cells and three hours after stimulation with K562 cells at a ratio of 1:1 as previously described (7). K562 (ATCC, CCL‐243) cells were used as a target cell line. NK cells were cultured in a medium containing 600 U mL^−1^ IL‐2 (Peprotech, USA) for 48 h to assess the degranulation of activated NK cells. CTL degranulation was evaluated in T lymphocytes 48 h after stimulation with 1.25 mg mL^−1^ 1‐ phytohemagglutinin‐L (PHA‐L, Vector, Germany) and 200 U mL^−1^ IL‐2 (Peprotech, USA). CTL degranulation was calculated by the difference in the median fluorescence intensity of CD107a of CTLs stimulated with CD3/CD28‐coated microbeads (Thermo Fisher Scientific, USA) at a ratio of 1:10 for 3 h and medium cultured cells. Degranulated in CD8^+^ T cells were stained with anti‐CD3‐FITC (clone *HIT3α*, BioLegend, USA), anti‐CD8‐PE‐Cy7 (clone *SK1*, BioLegend, USA), and anti‐CD107a‐APC (clone *H4A3*, BioLegend, USA). Degranulated in NK cells were stained with anti‐CD3‐APC (clone *HIT3α*, BioLegend, USA), anti‐CD56‐BV421 (clone *HCD56*, BioLegend, USA), and anti‐CD107a‐FITC (clone *H4A3*, BioLegend, USA) antibodies.

***NK cell cytotoxicity test***

The assay conditions were optimized by incubating EGFP‐K562 (target) cells with PBMCs (effector cells) obtained from patients, healthy donors and mother for the NK cell cytotoxicity assay. The test was performed as previously described (8, 9). Briefly, PBMCs were resuspended at 2 × 10^6^ mL−1, while EGFP‐K562 cells were adjusted to 2 × 10^6^ /mL in complete medium (1640 + 10% FBS). Effector (E) cells from PBMCs and target (T) cells (100 μL each) were mixed at a ratio of 1:1. Moreover, only EGFP‐K562 cells were included as the background control. Then, the samples were incubated at 37°C with 5% CO2 for 2 h. The optimized assay conditions were applied in subsequent experiments. The samples were transferred to flow cytometry tubes following incubation. After removal of the culture medium by centrifugation, the cells were sequentially washed with cold phosphate-buffered saline (PBS, Thermo Fisher Scientific, USA) and binding buffer (Annexin V‐PE Apoptosis Detection Kit, eBioscience, USA). Then, the cells were resuspended in 100 μl binding buffer containing 5 μl of Annexin V‐PE, gently mixed, and incubated in the dark for 15 min at room temperature. Afterward, 5 μl of (Annexin V‐PE Apoptosis Detection Kit, eBioscience, USA) was added before detection by flow cytometry. The proportions of cells in the EGFP‐K562 gate, including live, early apoptotic, late apoptotic and necrotic cells, were determined. After coculture of effector cells with target cells, the apoptosis ratio of target cells was considered to reflect NK cell cytotoxicity. Moreover, untreated EGFP‐K562 cells were stained with Annexin V‐Percp/propidium iodide (PI) as controls to assess naturally occurring apoptosis. The NK cell activity (%) was calculated as the apoptosis ratio of target cells in E/T coculture divided by the natural apoptosis ratio of target cells only.

***CTL cytotoxicity test***

CTL cytotoxicity was calculated by the percentage of cytotoxic effects on the target cells. The concentration of the PBMCs was adjusted to 2×10^6^/mL, and Dynabeads™ Human T-Activator CD3/CD28 magnetic beads were added. After six days of culture, effector cells from PBMCs and target cells were mixed at ratios of 3:1, 1:1, and 1:3. Moreover, PBMCs were included as the background control, and three replicate wells were set up for each effector ratio. Luciferase-labeled Nalm6 cells (Nalm6-Luci) were used as target cells. After 24 h of coculture of target and effector cells, the luciferase cleavage substrate (Steady-Glo® Luciferase Assay System, Promega, USA) was added, and the absorbance was measured using Synergy 2.

## Supplementary References

1. Zhang P, Yang X, Cao Y, Wang J, Zhou M, Chen L, et al. Autologous stem cell transplantation in tandem with Anti-CD30 CAR T-cell infusion in relapsed/refractory CD30(+) lymphoma. Exp Hematol Oncol. 2022;11(1):72.

2. Wang D, Zeng C, Xu B, Xu JH, Wang J, Jiang LJ, et al. Anti-CD30 chimeric antigen receptor T cell therapy for relapsed/refractory CD30(+) lymphoma patients. Blood Cancer J. 2020;10(1):8.

3. Rossi J, Paczkowski P, Shen YW, Morse K, Flynn B, Kaiser A, et al. Preinfusion polyfunctional anti-CD19 chimeric antigen receptor T cells are associated with clinical outcomes in NHL. Blood. 2018;132(8):804-14.

4. Wang N, Hu X, Cao W, Li C, Xiao Y, Cao Y, et al. Efficacy and safety of CAR19/22 T-cell cocktail therapy in patients with refractory/relapsed B-cell malignancies. Blood. 2020;135(1):17-27.

5. Zhang W, Yang L, Guan YQ, Shen KF, Zhang ML, Cai HD, et al. Novel bioinformatic classification system for genetic signatures identification in diffuse large B-cell lymphoma. BMC Cancer. 2020;20(1):714.

6. Shen K, Wang J, Zhou K, Mu W, Zhang M, Deng X, et al. CD137 deficiency because of two novel biallelic TNFRSF9 mutations in a patient presenting with severe EBV-associated lymphoproliferative disease. Clin Transl Immunology. 2023;12(5):e1448.

7. Bryceson YT, Pende D, Maul-Pavicic A, Gilmour KC, Ufheil H, Vraetz T, et al. A prospective evaluation of degranulation assays in the rapid diagnosis of familial hemophagocytic syndromes. Blood. 2012;119(12):2754-63.

8. Zhang J, Wang Y, Wu L, Wang J, Tang R, Li S, et al. Application of an improved flow cytometry-based NK cell activity assay in adult hemophagocytic lymphohistiocytosis. Int J Hematol. 2017;105(6):828-34.

9. Gao L, Dang X, Huang L, Zhu L, Fang M, Zhang J, et al. Search for the potential "second-hit" mechanism underlying the onset of familial hemophagocytic lymphohistiocytosis type 2 by whole-exome sequencing analysis. Transl Res. 2016;170:26-39.
